# Distribution and Inhibition of Liposomes on *Staphylococcus aureus* and *Pseudomonas aeruginosa* Biofilm

**DOI:** 10.1371/journal.pone.0131806

**Published:** 2015-06-30

**Authors:** Dong Dong, Nicky Thomas, Benjamin Thierry, Sarah Vreugde, Clive A. Prestidge, Peter-John Wormald

**Affiliations:** 1 Department of Surgery- Otorhinolaryngology Head and Neck Surgery, The Queen Elizabeth Hospital, and the University of Adelaide, Adelaide, South Australia; 2 Ian Wark Research Institute, University of South Australia, Mawson Lakes, South Australia; 3 The Rhinology Department, the First Affiliated Hospital of Zhengzhou University, Zhengzhou, China; University of Malaya, MALAYSIA

## Abstract

**Background:**

*Staphylococcus aureus* and *Pseudomonas aeruginosa* are major pathogens in chronic rhinosinusitis (CRS) and their biofilms have been associated with poorer postsurgical outcomes. This study investigated the distribution and anti-biofilm effect of cationic (+) and anionic (-) phospholipid liposomes with different sizes (unilamellar and multilamellar vesicle, ULV and MLV respectively) on *S*. *aureus* and *P*. *aeruginosa* biofilms.

**Method:**

Specific biofilm models for *S*. *aureus* ATCC 25923 and *P*. *aeruginosa* ATCC 15692 were established. Liposomal distribution was determined by observing SYTO9 stained biofilm exposed to DiI labeled liposomes using confocal scanning laser microscopy, followed by quantitative image analysis. The anti-biofilm efficacy study was carried out by using the alamarBlue assay to test the relative viability of biofilm treated with various liposomes for 24 hours and five minutes.

**Results:**

The smaller ULVs penetrated better than larger MLVs in both *S*. *aureus* and *P*. *aeruginosa* biofilm. Except that +ULV and –ULV displayed similar distribution in *S*. *aureus* biofilm, the cationic liposomes adhered better than their anionic counterparts. Biofilm growth was inhibited at 24-hour and five-minute exposure time, although the decrease of viability for *P*. *aeruginosa* biofilm after liposomal treatment did not reach statistical significance.

**Conclusion:**

The distribution and anti-biofilm effects of cationic and anionic liposomes of different sizes differed in *S*. *aureus* and *P*. *aeruginosa* biofilms. Reducing the liposome size and formulating liposomes as positively charged enhanced the penetration and inhibition of *S*. *aureus* and *P*. *aeruginosa* biofilms.

## Introduction

Both *Staphylococcus aureus* and *Pseudomonas aeruginosa* are major pathogens causing community-acquired and nosocomial infections all over the world. In the field of rhinology, evidence from molecular diagnostics in a recent microbiome study demonstrated that the prevalence of *S*. *aureus* and *P*. *aeruginosa* in patients of chronic rhinosinusitis (CRS) was 61% and 8% respectively[1].

Bacterial biofilms are highly organized structures consisting of bacterial communities and extracellular matrix[2]. Their presence in CRS patients has been associated with decreased quality of life, greater mucosal inflammation, more severe osteitis and higher incidence of recurrent infection[2–5]. In particular, amongst the most frequently isolated pathogens from nasal mucosa of CRS patients, *S*. *aureus* biofilm plays a predominant role in negatively influencing postoperative outcomes[6]and *P*. *aeruginosa* biofilm has been linked to poorer postsurgical progression as well[7].

When biofilm is formed, bacteria become up to 1000 times more resistant to antibiotic treatment compared to the planktonic type, which makes eradication even more challenging[8, 9]. It has been proven that liposome-encapsulation improves the efficacy of various antibacterial and antifungal drugs against a broad range of pathogens *in vitro* and *in vivo*[10–15], by more effective drug delivery. Due to their biocompatibility, liposomes formulated by ubiquitous and nontoxic lipid-like molecules are promising in preventing biofilm formation and removing existing biofilm[16–18]. Based on an increasing number of studies on the interaction between liposomes and biofilms, the size and charge of the liposome are considered as important factors influencing penetration[18–20]. Depending on the bacteria species the heterogeneous composition of biofilm matrix and the cell membranes could also affect the interaction between bacteria and liposomes as well as their therapeutic efficacy[21]. To date, how the liposomal properties such as the charge, size and structure influence this interaction, and how this correlates with the anti-biofilm efficacy is not fully understood.

The goal of this study was to investigate the distribution and anti-biofilm effects of cationic and anionic liposomes with various sizes on *S*. *aureus* and *P*. *aeruginosa* biofilm in vitro. To this end we prepared a library of phospholipid liposomes and determined the liposome-biofilm interaction using confocal scanning laser microscopy (CSLM). The viability of biofilms after liposomal intervention was determined using the alamarBlue assay.

## Materials and Methods

### Liposome Preparation

Based on the well-documented liposome preparation method originated by Bangham et al[22, 23], the multilamellar vesicles (MLV) and unilamellar vesicles (ULV) were produced as follows: 1,2-distearoyl-sn-glycero-3-phosphocholine (DSPC) and 1,2-dipalmitoyl-sn-glycero-3-phosphoglycerol sodium salt (DPPG) were obtained from Lipoid (Lipoid GmbH, Ludwigshafen, Germany). 1,2-dioleyl-3-trimethylammonium-propane (DOTAP) was purchased from Avanti Polar Lipids (Montgomery, AL, USA). 1,1'-dioctadecyl-3,3,3',3'-tetramethylindocarbocyanine perchlorate (DiI), chloroform, and methanol were purchased from Sigma (Sigma-Aldrich, Sydney, Australia). All solvents were of analytical grade and were used as received.

The liposomes were prepared by the lipid-film hydration method described previously[8, 24]. Briefly, phospholipids (DSCP/DPPG and DSCP/DOTAP) were used in a 4:1 molar ratio for the preparation of anionic and cationic liposomes, respectively. The phospholipids were dissolved in 10 mL of a mixture of chloroform/ methanol (90: 10%, vol/vol) in a 50 mL round bottom flask. The organic solvent was removed overnight under vacuum on a rotavapor (Buechi Labortechnik, Flawil Switzerland). The resulting dry lipid film was then rehydrated for 2 hours with 10 ml of a sterile physiological saline solution to produce a liposome dispersion consisting of MLV. ULVs were prepared from MLV dispersions via extrusion using a thermo extruder (Northern Lipids, Burnaby, BC, Canada) at 60°C (i.e. 5°C above the transition temperature of DSPC). The liposome dispersions were subjected to ten consecutive extrusion cycles through two stacked polycarbonate membranes (Millipore, Billerica, MA, USA) with a pore size of 1000 nm, followed by another ten consecutive extrusion cycles using two stacked membranes of 200 nm pore-size. The final lipid concentration of the MLV and ULV dispersion was 25 mM. An analogous protocol[25, 26] was employed for the preparation of fluorescently labeled liposomes for the confocal microscopy studies by addition 0.2% (w/w) of the lipophilic membrane stain DiI to the organic solvent/lipid mixture.

### Liposome Characterization

The MLV and ULV dispersions were characterised for particle size, particle size distribution, and zeta-potential by dynamic light scattering (DLS) and phase analysis light scattering (PALS) at 37°C using a Zetasizer Nano-ZS particle sizer (Malvern, Worcestershire, UK). Dynamic light scattering follows the time-dependent fluctuation of the Rayleigh scattering of dispersed particles resulting from Brownian motion. Application of the Mie theory and Stokes-Einstein equation allows the calculation of the particle size and the width of the size distribution from the time-autocorrelation function. Phase analysis light scattering is based on the Smoluchowski/ Huckel theories using the electrophoretic mobility of the particles (i.e. the ratio of the velocity of particles to the field strength) to compute the mean zeta potential. Samples were prepared by Dilution of 0.1 mL of the dispersions with 0.9 mL of Milli-Q water. All measurements were carried out in triplicates and the results are reported as the mean ± standard deviation.

### Bacterial Strains and Biofilm Formation

The known biofilm forming strains *S*. *aureus* American Type Culture Collection (ATCC) 25923 and *P*. *aeruginosa* ATCC 15692 were used in the current study. The bacterial cultures for both of the strains were established as previously published[8]with modifications. Briefly, bacterial strains from the frozen glycerol stock were streaked on the nutrient agar (Oxoid, SA, Australia) plate and incubated at 37°C overnight to revive. Then bacterial suspension of 1 McFarland unit in 0.9% saline was adjusted with single colonies from the plate culture above. The suspension was then diluted to 1:15 in CSF broth (Thermo Fisher, SA, Australia) to be used in the formation of biofilms.

Different biofilm models were established for the liposomal distribution and anti-biofilm studies separately with optimized growth conditions. To observe the distribution of liposomes in biofilm using CSLM, 8-well culture slides (BD Falcon, NSW, Australia) were used. For *S*. *aureus* biofilm formation, 300μl of the bacterial suspension was added to each well and incubated at 37°C on the gyrorotary shaker (Ratek, Vic, Australia) at 70rpm. After 24-hour incubation, all the media was gently replaced by fresh CSF broth to maintain bacterial viability, and then followed by another 48-hour incubation at 37°C with 5% CO_2_ and 90% humidity to allow further biofilm formation. For *P*. *aeruginosa*, the culture slide was adjusted to sit at 45° angle from horizontal with 150μl of the aforementioned bacterial suspension in each well for biofilm formation at the air-liquid interface[27]. After 24-hour incubation at 37°C statically, 50μl media was carefully replaced and then followed by another 48-hour culture in the same condition as for the *S*. *aureus* biofilm.

For the liposomal anti-biofilm experiments, the method of Kien[9] and Jardeleza[8] was used with the only modification that 150μl of *S*. *aureus* or *P*. *aeruginosa* dilution was pipetted into the wells of the 96 well clear-bottom microplates (Corning Life Sciences Plastic, NY, USA) and incubated for 48 hours at 37°C on the gyrorotary shaker at 70rpm for biofilm growth.

### Liposomal Distribution Study

The liposomal distribution study was conducted within 20 hours after the DiI-labelled liposomes were prepared. The bacterial biofilm on the culture slide was rinsed twice with saline (0.9% NaCl) to remove the planktonic cells[19, 28] and then incubated with 300μL/well of the DiI-labelled liposomes for five minutes. Saline was applied as the non-treatment control. Subsequently, the samples were fixed with 300μL/well of 5% glutaraldehyde (Sigma Aldrich, St Louis, MO, USA) for 30 minutes at room temperature prior to staining. Then 300μL/well of 5μM SYTO9 (Invitrogen Molecular Probes, Vic, Australia) solution in saline was inoculated to each well and incubated in the dark for 15 minutes at room temperature to label the cells. Every single step was followed by two saline rinses. After removing the upper chamber of the culture slide, the samples were sealed with glycerol (Sigma-Aldrich, Sydney, Australia) and ready to be examined under the confocal scanning laser microscope. For *S*. *aureus* biofilm, the Leica TCS SP5 (Leica Microsystems, Wetzlar, Germany) was employed with the settings as follows: 63×/1.2 objective and 0.5μm for laser scanning step size. Fluorescence from DiI was detected using the excitation wavelength of 561 nm and emission of 570–600 nm; fluorescence from SYTO9 was excited at 476 nm and collected at 500–520 nm. The *P*. *aeruginosa* biofilm study was carried out using a Zeiss LSM700 confocal scanning laser microscope (Carl Zeiss Microscopy GmbH, Oberkochen, Germany), where the settings were adjusted slightly due to the different hardware: 63×/1.4 objective, and the excitation/ emission wavelength for DiI and SYTO9 was 555nm/ 570nm~ and 488nm/ ~520nm respectively. For each sample, three representative *z*-stacks containing the full thickness of biofilm with liposomes were captured and experiments were repeated twice. The image processing and analysis were carried out by the software ZEN (ZEISS Microscope Software 2012) and ImageJ 1.43 (Wayne Rasband, National Institutes of Health, Bethesda, MD, USA).

### Liposomal Anti-biofilm Study

The liposomal anti-biofilm study was conducted within 20 hours after the liposomes were prepared. The prepared biofilm-coated wells of the 96-well microtiter plate were rinsed twice in saline to remove planktonic bacteria and then exposed to 200μL liposome dispersion for five minutes and 24 hours at 37°C followed by another two washes. After treatment, 200μL CSF broth was pipetted to each well and incubated at 37°C for 24 hours to allow the biofilm recovery with adhered liposomes[8]. Subsequently, after the effect of liposomes to alamarBlue (Invitrogen, CA, USA) was confirmed as negligible (S1 Fig), the alamarBlue assay (Invitrogen, CA, USA) was performed to test the viability of challenged biofilm. According to the manufacturer’s instructions, the alamarBlue dye was diluted to 1:10 in CSF broth. After two rinses, 250μL/well of the alamarBlue dilution was added and incubated statically at 37°C for one hour. Finally, the fluorescence intensity from the samples was measured by FLUOstar OPTIMA plate reader (BMG Labtech, Vic, Australia) equipped with an excitation filter of 520–540 nm and an emission filter of 580–600 nm. In our experiment, saline was used as the negative control for the liposomal treatment and the wells that did not contain biofilm were stained as the background. All treatments were carried out in quadruplicate and the experiments repeated twice.

### Statistical Analysis

Kruskal-Wallis test with Mann-Whitney test for post-hoc comparisons was carried out by Graphpad Prism 5.0 (San Diego, CA, USA) for analyzing differences among selected pairwise treatment groups. A *P* value of < 0.05 was considered as statistically significant.

## Results

### Liposome Characterization

The particle size (reported as the intensity weighted z-average), particle size distribution (reported as polydispersity index, PDI), and zeta-potential were summarized in Table 1. Without further processing the initially produced anionic and cationic MLV dispersions appeared milky-white and showed high PDI (>0.6) which did not allow accurate size measurements of the MLV dispersions by DLS. The extrusion of anionic and cationic MLV generated uniform ULV of approximately 130–140 nm with a narrow particle size distribution (PDI≤0.1). As expected, the inclusion of charged lipids (DPPG and DOTAP for anionic and cationic liposomes, respectively) resulted in negative and positive zeta-potentials of the particles (absolute zeta values of approximately 60–90 mV) indicating sufficient colloidal stability for all the produced dispersions for the duration of the study.

**Table 1:** Liposome characterization.

Liposome formulation	Size (nm)	PDI	Zeta-potential (mV)
-MLV	>1000	0.68 ± 0.26	-70.8 ± 2.3
-ULV	141.0 ± 1.3	0.09 ± 0.01	-56.6 ± 0.8
+MLV	>1000	>0.86	+89.1± 4.2
+ULV	128.6 ± 2.3	0.12 ± 0.02	+58.6 ± 1.3

Data are expressed as mean ± SD, n = 3.

PDI = polydispersity index;-MLV = anionic multilamellar vesicle;-ULV = anionic unilamellar vesicle; +MLV = cationic multilamellar vesicle; +ULV = cationic unilamellar vesicle.

### Liposomal Distribution

**Fig 1:**
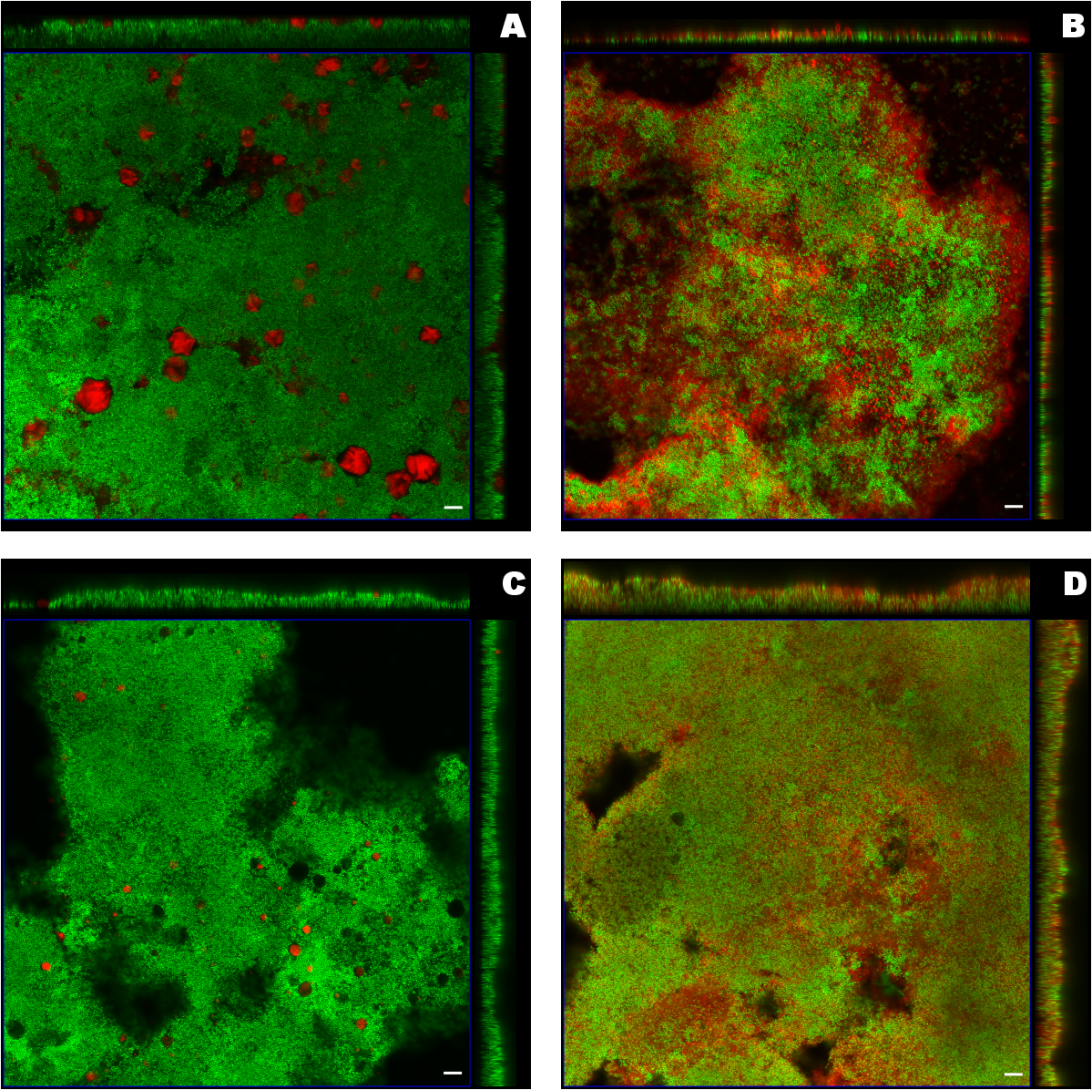
Orthogonal images from confocal laser scanning microscopy of *S*. *aureus* biofilm treated with liposomes. Liposomes including A) cationic multilamellar vesicle (+MLV), B) cationic unilamellar vesicle (+ULV), C) anionic multilamellar vesicle (-MLV) and D) anionic unilamellar vesicle (-ULV), were labeled red (DiI) and biofilm was stained green (SYTO-9). The middle square image in the blue frame was the 3D projection of five layers in the biofilm centre from the *z*-stack with the mode of maximum intensity, including the layer with the maximum integrated fluorescent density of the green channel. The images on the top and right side were the central layer from the y-stack and x-stack respectively. The scale bar represented 10μm.

**Fig 2:**
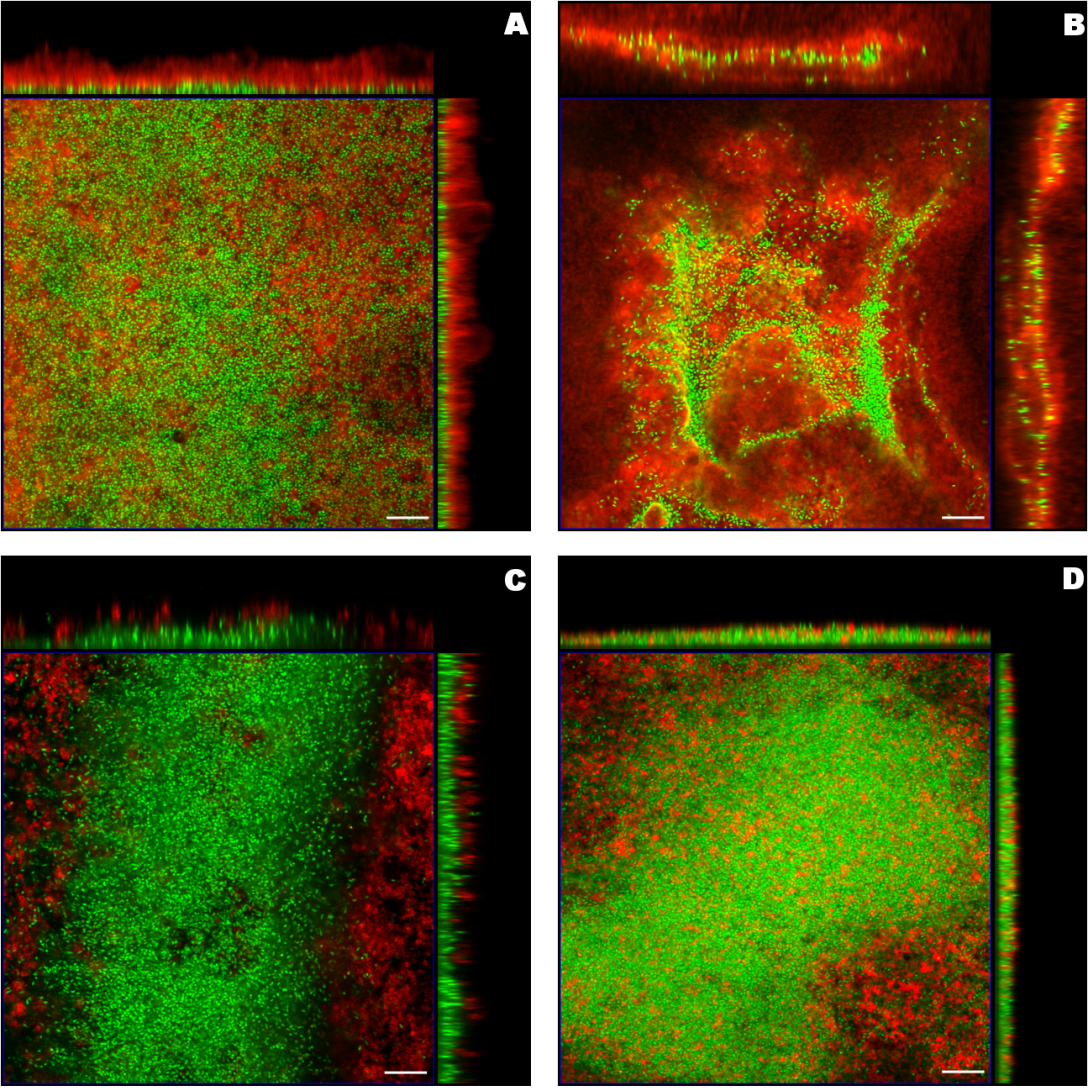
Orthogonal images from confocal laser scanning microscopy of *P*. *aeruginosa* biofilm treated with liposomes. Liposomes including A) cationic multilamellar vesicle (+MLV), B) cationic unilamellar vesicle (+ULV), C) anionic multilamellar vesicle (-MLV) and D) anionic unilamellar vesicle (-ULV), were labeled red (DiI) and biofilm was stained green (SYTO-9). The middle square image in the blue frame was the 3D projection of two layers in the biofilm centre from the *z*-stack with the mode of maximum intensity, including the layer with the maximum integrated fluorescent density of the green channel. The images on the top and right side were the central layer from the y-stack and x-stack respectively. The scale bar represented 10μm.

The formulations and bacteria were presented in red and green respectively, reflecting the employed fluorescent dyes, DiI and SYTO9. Due to the differences of the microscopy settings and the biofilm thickness, five layers in the centre of *S*. *aureus* biofilm from the entire *z*-stack and two layers in the centre of *P*. *aeruginosa* biofilm were chosen as the representative projected images. The z-projections along with the cross-sections from the center of the x-stack and y-stack exhibited different distributions of liposomes in biofilm (Fig 1 and Fig 2). For liposomes in distinct sizes, the larger liposomes (+MLV and-MLV) appeared like clouds floating above both *S*. *aureus* and *P*. *aeruginosa* biofilm. On the other hand, as expected, the smaller liposomes (+ULV and-ULV) penetrated biofilm better than the corresponding larger ones (see x- and y-stacks in Fig 1B and 1D and Fig 2B and 2D, depicting deep penetration of ULVs within the biofilm, compared to x- and y-stacks in Fig 1A and 1C and Fig 2A and 2C, showing MLVs in larger aggregations on top and around the biofilms). In terms of the liposome charge, more cationic liposomes (+MLV and +ULV) attached to *P*. *aeruginosa* biofilms compared with their anionic counterparts (Fig 2A and 2B compared to Fig 2C and 2D). Also, after exposure to +ULV, the appearance of *P*. *aeruginosa* biofilm was altered from a plateau-shaped structure to a dispersed multi-laminar structure (see Fig 2B). The changes due to the different charges were not as obvious in *S*. *aureus* biofilm as in *P*. *aeruginosa* biofilm.

**Fig 3:**
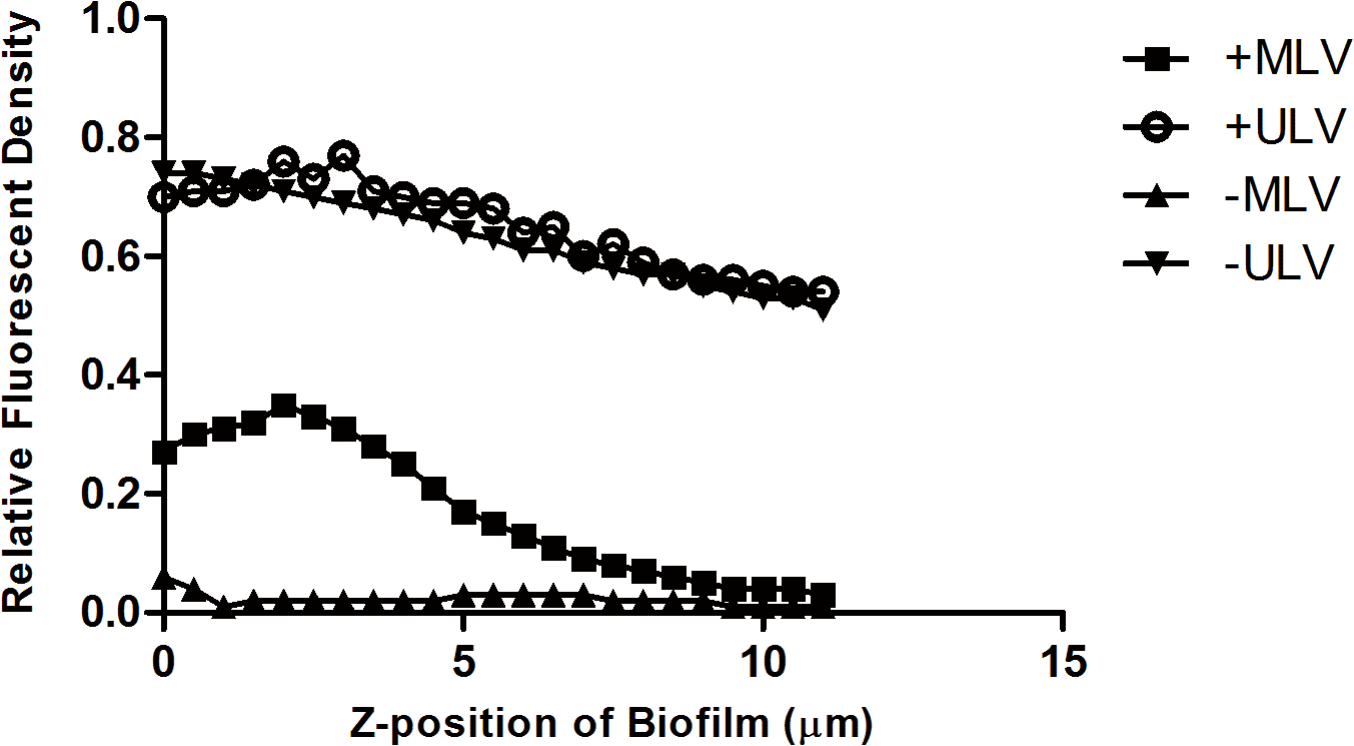
Liposomal distribution in *S*. *aureus* biofilm. The *y-*axis represents the integrated fluorescent density of the red channel (liposomes) normalized by that of the green channel (biofilm), reflecting the amount of liposomes per unit of biomass in each layer of the biofilm. The *x-*axis indicates the z-position of the layers in biofilm, where the location of the top surface of biofilm was assigned as zero. +MLV: cationic multilamellar vesicle; +ULV: cationic unilamellar vesicle;-MLV: anionic multilamellar vesicle;-ULV: anionic unilamellar vesicle.

**Fig 4:**
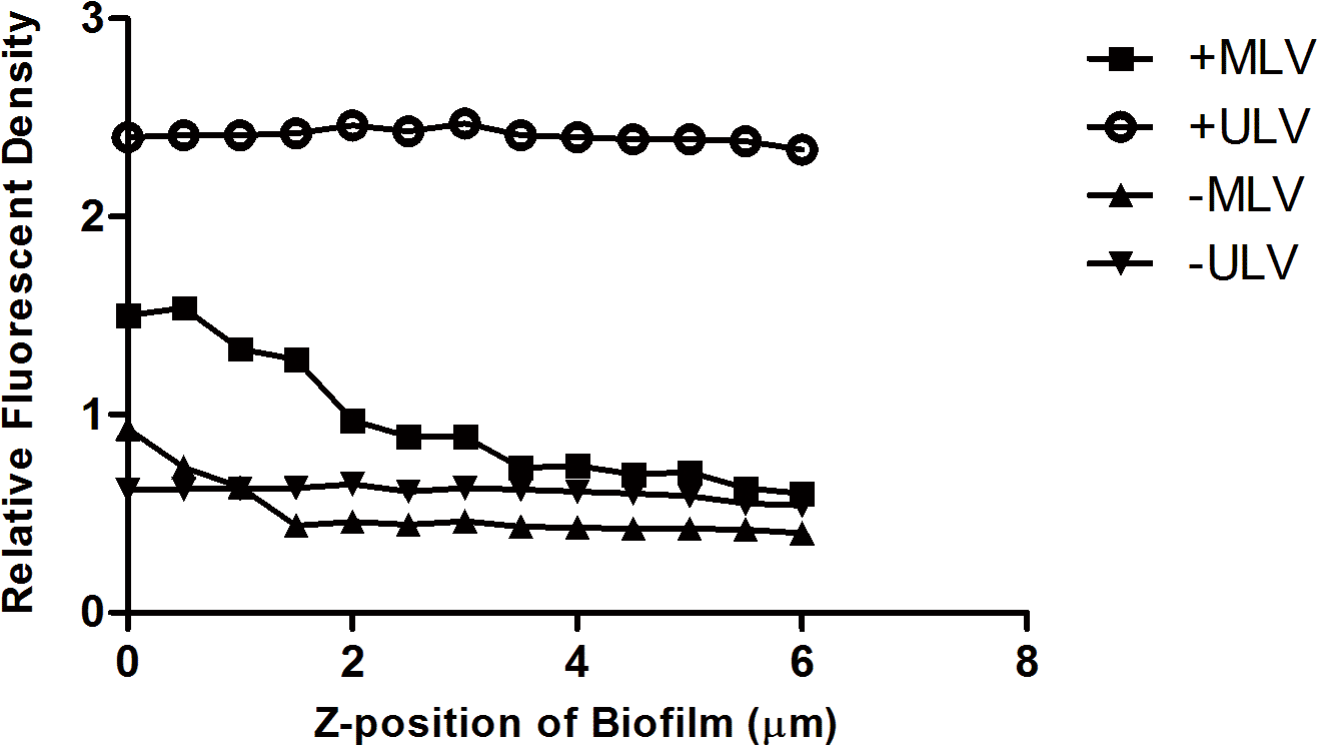
Liposomal distribution in *P*. *aeruginosa* biofilm. The *y-*axis represents the integrated fluorescent density of the red channel (liposomes) normalized by that of the green channel (biofilm), reflecting the amount of liposomes per unit of biomass in each layer of the biofilm. The *x-*axis indicates the *z*-position of the layers in biofilm, where the location of the top surface of biofilm was assigned as zero. +MLV: cationic multilamellar vesicle; +ULV: cationic unilamellar vesicle;-MLV: anionic multilamellar vesicle;-ULV: anionic unilamellar vesicle.

In order to make a quantitative comparison for the distribution of the four different liposomes in the biofilms, the integrated fluorescent density of every single layer in each *z*-stack was measured separately for the red and green channel. The integrated fluorescent density of the red channel was normalized by that of the green channel, as shown in Fig 3 and Fig 4, reflecting the distribution of liposomes per unit of biomass in each layer from the surface to the base of the biofilm. The mean thickness of *S*. *aureus* biofilm was 12μm while that of *P*. *aeruginosa* biofilm was 6.5μm in the present study, so the normalized integrated fluorescent density of the red channel was calculated on 24 and 13 layers in total to demonstrate the liposomal distribution in the entire *z*-stack for *S*. *aureus* and *P*. *aeruginosa* biofilm respectively. It was clear that for both *S*. *aureus* and *P*. *aeruginosa* biofilm,–MLV did not associate strongly with the body of the biofilms, and that +MLV was mainly found in the upper part of the biofilm structure. The distributions of ULVs in *S*. *aureus* biofilm were similar to each other. However, +ULV displayed higher normalized fluorescent density in the whole *z*-stack of *P*. *aeruginosa* biofilm than–ULV.

**Fig 5:**
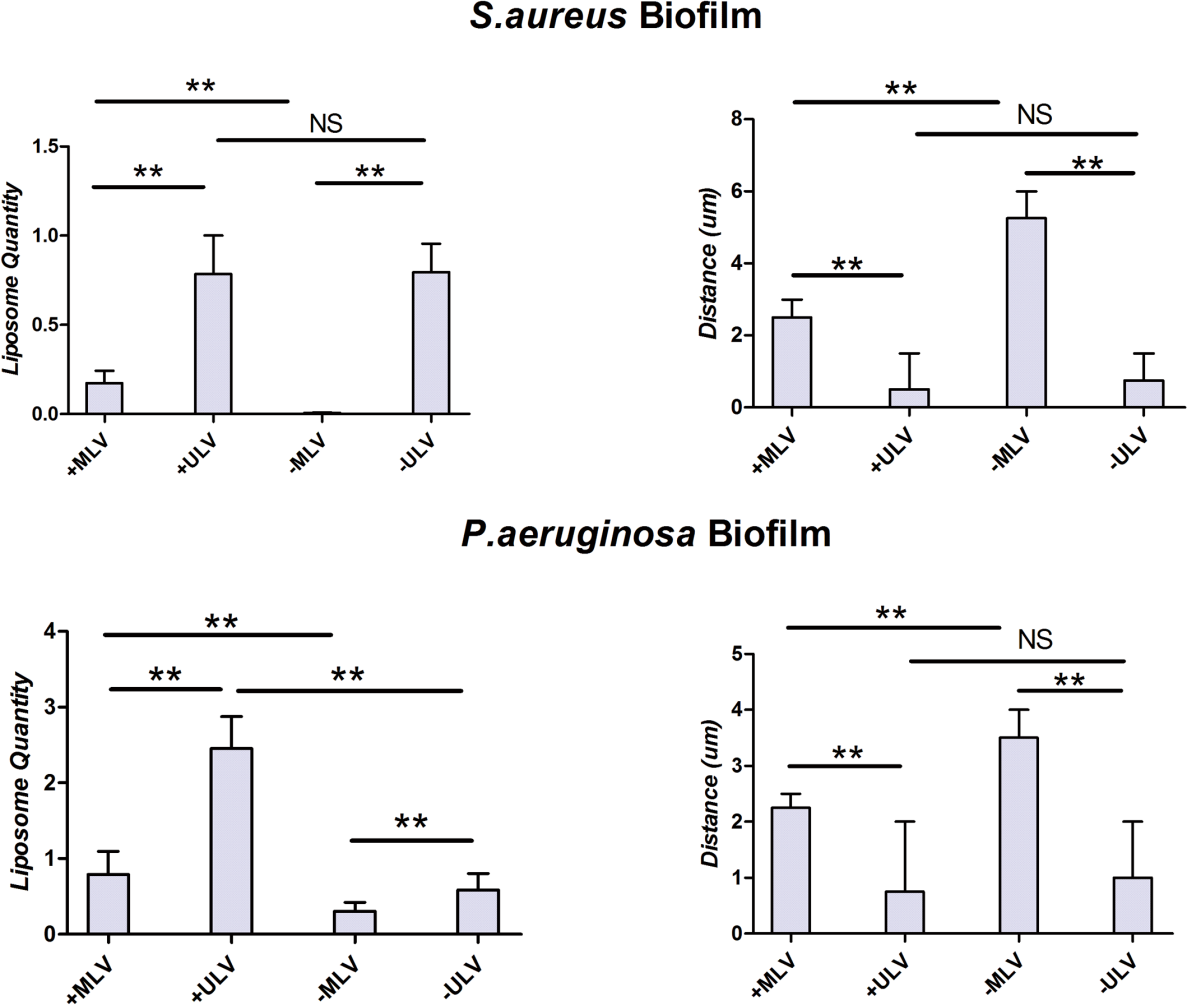
Comparison of liposomal distribution in *S*. *aureus* and *P*. *aeruginosa* biofilms. Comparison of liposomal distribution in both *S*. *aureus* and *P*. *aeruginosa* biofilms measured by *Liposome Quantity*, reflecting the amount of liposomes per unit of biomass in the most intensive section of biofilm, and *Distance*, representing how close the majority of liposome approached to the core of biofilm. The histogram displayed medians with ranges, and the comparison was carried out by Kruskal-Wallis test with Mann-Whitney test for post-hoc. **: *P*<0.01; NS: no statistic significance, *P*>0.05; +MLV: cationic multilamellar vesicle; +ULV: cationic unilamellar vesicle;-MLV: anionic multilamellar vesicle;-ULV: anionic unilamellar vesicle.

After the values of integrated fluorescent density for both channels in the entire *z*-stack were recorded, the layers with the maximum values were picked, representing the location where the maximum amount of liposomes or biofilm biomass appeared (Maxred and Maxgreen, respectively). Thus the following two parameters were designed to make further statistical comparison in our study: First, the *Liposome Quantity*, which was the integrated fluorescent density of the red channel on the Max_green_ layer and normalized by it, reflecting the distribution of liposomes per unit of biomass in the most intensive section (the “core” or the centre) of the biofilm. Second, the *Distance* between the layer of Max_red_ and Max_green_, representing the proximity of the majority of the liposomes to the core of the biofilm. Data were summarized in Table 2 and Fig 5.

**Table 2:** Distribution of liposomes in *S*. *aureus* and *P*. *aeruginosa* biofilm.

	*Liposome Quantity*					*Distance*				
	+MLV	+ULV	-MLV	-ULV	*P* value	+MLV	+ULV	-MLV	-ULV	*P* value
*S*. *aureus*	0.1735[0.1277–0.2426]	0.7855[0.6335–1.001]	0.006651[0.005731–0.009910]	0.7953[0.5872–0.9562]	0.0002	2.50[2.00–3.00]	0.50[0–1.50]	5.25[4.50–6.00]	0.75[0–1.50]	0.0002
*P*. *aeruginosa*	0.7933[0.6656–1.094]	2.452[2.380–2.874]	0.3047[0.2806–0.4230]	0.5850[0.4861–0.8029]	0.0001	2.25[2.00–2.50]	0.75[0.50–2.00]	3.50[3.00–4.00]	1.00[0.50–2.00]	0.0003

Data are expressed as median and range, n = 6.

All *P* values are determined with Kruskal-Wallis test.

+MLV = cationic multilamellar vesicle; +ULV = cationic unilamellar vesicle;-MLV = anionic multilamellar vesicle;-ULV = anionic unilamellar vesicle.

By determining the *Liposome Quantity* and the *Distance*, the interactions of the liposomes with the biofilms were compared based on the liposomal size and charge. There was significantly larger amounts of smaller liposomes adhering to both *S*. *aureus* (+MLV vs +ULV, *P* = 0.0022;-MLV vs-ULV, *P* = 0.0022) and *P*. *aeruginosa* biofilm (+MLV vs +ULV, *P* = 0.0022;-MLV vs-ULV, *P* = 0.0022) than their larger counterparts with the same charge. The smaller liposomes were also able to get significantly closer to the center of the biofilms (*S*. *aureus*, +MLV vs +ULV: *P* = 0.0047;-MLV vs-ULV, *P* = 0.0049; *P*. *aeruginosa*: +MLV vs +ULV, *P* = 0.0086;-MLV vs-ULV, *P* = 0.0047). As to liposomes with opposite charges, +MLV showed a significantly higher *Liposome Quantity* (*S*. *aureus*: *P* = 0.0022; *P*. *aeruginosa*: *P* = 0.0022) and shorter *Distance* between Max_red_ and Max_green_ (*S*. *aureus*: *P* = 0.0047; *P*. *aeruginosa*: *P* = 0.0045) than–MLV for biofilms of both strains. When +ULV and–ULV were compared, there were no significant differences in either *Liposome Quantity* (*P* = 0.8182) or *Distance* between Max_red_ and Max_green_ (*P* = 0.6191) in *S*. *aureus* biofilm. However, the amount of +ULV co-localizing with *P*. *aeruginosa* biofilm was significantly greater than–ULV (*P* = 0.0022), and the *Distance* between Max_red_ and Max_green_ for +ULV was shorter than–ULV, although statistical significance was not reached (*P* = 0.5597).

### Liposomal Anti-biofilm Effect

**Fig 6:**
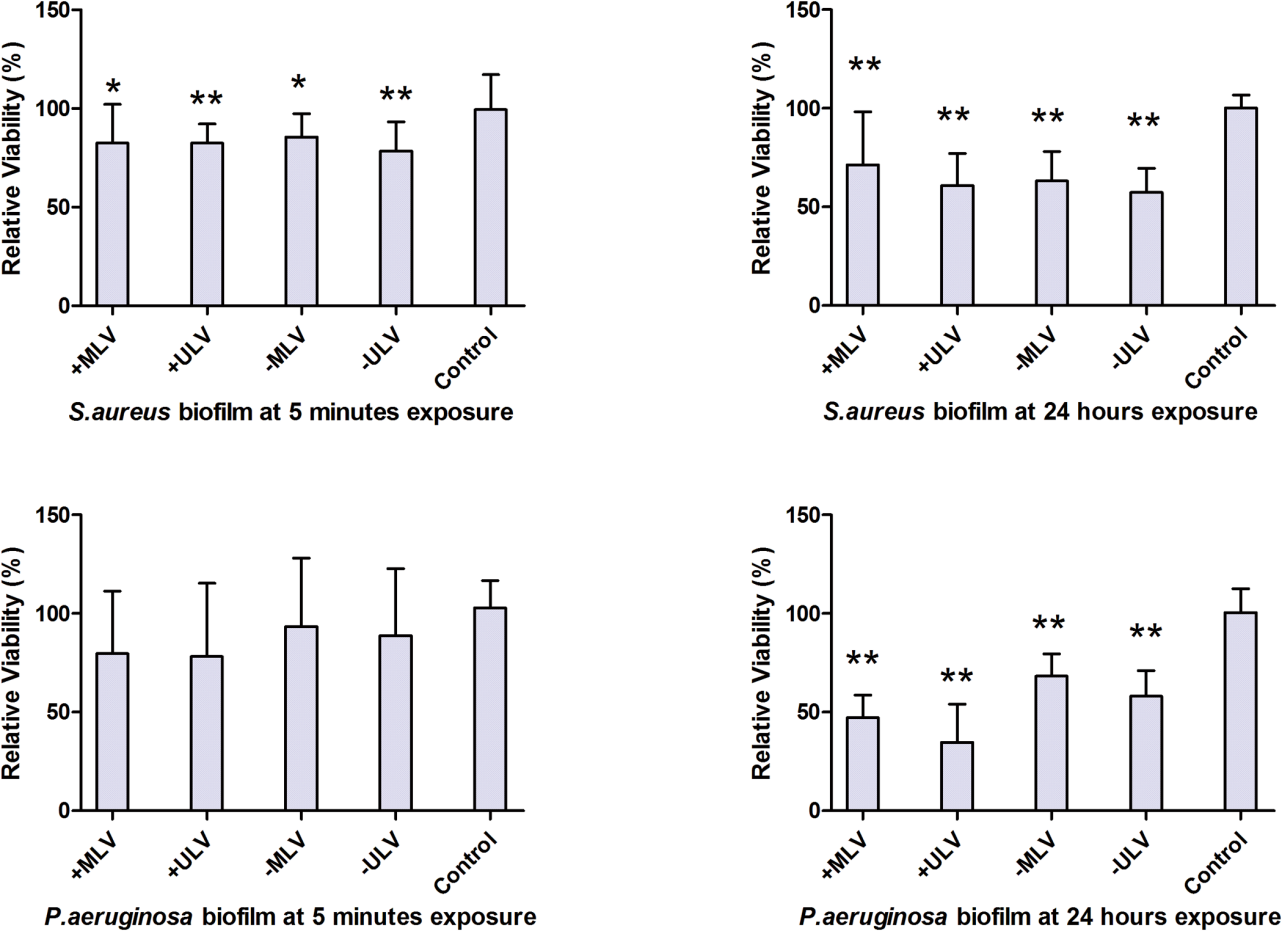
Comparison of anti-biofilm effect of liposomes against *S*. *aureus* and *P*. *aeruginosa* biofilms. Comparison of anti-biofilm effect of liposomes against both *S*. *aureus* and *P*. *aeruginosa* biofilms tested by alamarBlue assay. The histogram displayed medians with ranges, and the comparison was carried out by Kruskal-Wallis test with Mann-Whitney test for post-hoc. *: *P*<0.05; **: *P*<0.01, compared to the untreated control; +MLV: cationic multilamellar vesicle; +ULV: cationic unilamellar vesicle;-MLV: anionic multilamellar vesicle;-ULV: anionic unilamellar vesicle.

After normalization to the mean of fluorescent intensity for the control wells containing biofilms without liposomal treatment, the result of biofilm relative viability was summarized in Table 3 and Fig 6. The data demonstrated that after five-minute and 24-hour exposure, all tested liposomes inhibited the growth of both *S*. *aureus* and *P*. *aeruginosa* biofilms. However, the decrease in the viability of *P*. *aeruginosa* biofilm after five-minute treatment with the different liposomes did not reach statistical significance. Post-hoc pairwise comparisons showed that at 24-hour exposure, the cationic liposomes displayed significantly stronger anti-biofilm effect than the anionic ones on *P*. *aeruginosa* biofilm (+MLV vs–MLV: *P* = 0.0052, +ULV vs–ULV: *P*< 0.0001), and the smaller +ULV had stronger anti-biofilm effects than the larger +MLV (*P* = 0.0249). Although the differences between liposomes against *P*. *aeruginosa* biofilm for five minutes and against *S*. *aureus* biofilm for both five minutes and 24 hours had no statistical significance (all *P*>0.05), there was a trend that the anti-biofilm effect of smaller liposomes was stronger than the corresponding larger ones.

**Table 3:** Relative viability of *S*. *aureus* and *P*. *aeruginosa* biofilm at five-minute and 24-hour exposure.

	Relative viability (%) at five-minute exposure					Relative viability (%) at 24-hour exposure				
	+MLV	+ULV	-MLV	-ULV	*P* value	+MLV	+ULV	-MLV	-ULV	*P* value
*S*. *aureus*	82.39[58.79–102.1]	82.44[63.70–92.14]	85.40[71.93–97.36]	78.50[60.63–93.23]	0.0101	71.34[47.52–98.31]	60.86[41.08–77.21]	63.20[51.93–78.00]	57.29[37.38–69.51]	0.0004
*P*. *aeruginosa*	79.71[41.21–111.4]	78.17[55.40–115.4]	93.46[68.28–128.2]	88.81[67.47–122.8]	0.2723	47.15[39.22–58.67]	34.73[27.42–54.00]	68.27[45.64–79.58]	58.01[55.99–71.06]	< 0.0001

Data are expressed as median and range, n = 8.

All *P* values are determined with Kruskal-Wallis test.

+MLV = cationic multilamellar vesicle; +ULV = cationic unilamellar vesicle;-MLV = anionic multilamellar vesicle;-ULV = anionic unilamellar vesicle.

## Discussion

The present study systematically investigated the distribution and anti-biofilm effects of liposomes with opposite charges and different sizes on biofilms of the two important pathogens, *S*. *aureus* and *P*. *aeruginosa*. Our data demonstrated that although the interaction between liposomes and biofilms differed for each species of biofilm, the smaller and cationic liposomes penetrated better and inhibited both biofilms.

Based on the nanotechnology in our study, the cationic liposomes were prepared in comparable size to their anionic counterparts, which made it feasible to directly compare individual liposomal property, i.e. size or charge, separately. The result from the liposomal distribution study displayed that ULVs were able to reach to the centre of both *S*. *aureus* and *P*. *aeruginosa* biofilms while MLVs with the same charge did not, suggesting that the size of the liposome influenced penetration. According to the research on the penetration of liposomes and other nanoparticles into *Burkholderia multivorans* and *Pseudomonas aeruginosa* biofilms, Forier et al. also reported that the larger the particle diameter, the more the particles were excluded from the smaller channels within the biofilms[20]. Biofilms have many protective mechanisms including modified microbial gene expression, altered microenvironment of the bacterial colonies (i.e. pH changes and oxygen deficient zones), the local accumulation of enzymes degrading antibiotics but the structural complexity and mechanical stability provided by the matrix undoubtedly restricts drug diffusion and contributes to the antibiotic resistance [21, 29–31]. Our data demonstrated that there was only a limited amount of MLVs diffusing inside biofilms and that most of the larger liposomes were immobilized at the biofilm surface. It is important to note that larger liposomes are preferable in regard to their higher drug encapsulation of hydrophilic drugs[8, 32]. However, the benefit of an increase in size might be outweighed by a reduced liposome penetration into biofilms leading to a less effective local drug delivery.

Liposomes with opposite charges but similar sizes were also compared in the distribution study. The data showed that +MLV adhered better than–MLV to both *S*. *aureus* and *P*. *aeruginosa* biofilms. Gross *et al*. proved that for *S*. *aureus*, the component with highly negative charge in the cell wall, teichoic acid, played a key role in the biofilm formation[33, 34]. As for *P*. *aeruginosa*, the well-investigated virulence factor, i. e. polyanion exopolysaccharide alginate, is an important component of its biofilm as well[35, 36]. Consequently, considering these negatively charged components, the cell surface of these bacteria has overall a moderately negative net charge[33, 37–40]. Hence it can be hypothesized that repulsive electrostatic forces resulted in less binding between anionic liposome and biofilm. This makes the cationic liposome promising in targeted drug delivery for the treatment of bacterial biofilms[18].

In terms of ULVs, the distribution of +ULV and–ULV appeared similar in *S*. *aureus* biofilm, whereas disparate in *P*. *aeruginosa* biofilm. It seemed that +ULV not only penetrated into *P*. *aeruginosa* biofilm but also changed the structure of the biofilm by dispersing it. This observation is potentially important as therapeutic formulations able to compromise the highly organized ultrastructure of biofilm may render the bacterial cells more susceptible to standard antibiotics or other agents having similar effects[41]. Our results indicate that the interaction of liposomes with bacterial biofilms is complex and not simply dictated by the surface charge or the net charge of the biofilm. This could be partly attributed to the architectural difference between biofilms formed by different microbes. Based on the patterns in which biofilm was separated by interstitial voids and water channels, biofilm models were abstracted as stalked or irregular branching, mushroom-shaped, dense confluent structures and the mixture of the former[21]. Although direct knowledge of the internal forces and construction of biofilms is limited, it is possible that after diffusing via interstices and channels into the body of biofilm, +ULV may disrupt its inner electrostatic equilibrium and collapse the microcolonies that are not dense or robust enough. However, this requires further investigation.

Since the liposomes used in this study were neither prepared with bactericidal compounds nor loaded with antimicrobial agents, it is essential to observe their effect on biofilms. If the liposomes formulated by nontoxic lipid-like molecules showed no nutrient effect on the bacteria, they would be more promising candidates to incorporate bactericidal compounds in a future study. The influence of liposomes on the growth of biofilm was investigated up to 24 hours. In order to simulate the rapid exposure of topical douching in the sinuses in clinic, a short exposure of 5 minutes was also tested. The inhibition effect of all four liposomes on biofilms was confirmed by testing the relative viability of the biofilm. Data demonstrated that after 24 hours exposure, the biofilm growth was restricted by up to 43% and 75% for *S*. *aureus* and *P*. *aeruginosa* respectively. Furthermore, after 24 hours exposure, all the four liposomes decreased the relative viability of biofilms more than that at the five minute time point, suggesting that *S*. *aureus* and *P*. *aeruginosa* were not able to grow on exposure to the blank liposomes. The mechanisms of liposomal toxicity against bacteria remain unclear. Based on data from studies on other nanoparticles[18, 42, 43], it can be hypothesized that after liposomes attach and incorporate themselves into the biofilm structure and microbial membrane, they are able to convert the signaling pathways[16]and disrupt the integrity of the cell membrane. This may play an important role in inhibiting biofilm growth[44, 45].

Jardeleza *et al*.[8]reported that blank–ULV showed more pronounced anti-biofilm effect than–MLV at five-minute exposure. Although the same *S*. *aureus* strain and anionic liposomes were used, our study did not reproduce the same effectivity. The difference on the inhibition extent between the two researches may be due to different experimental methods, which were measured by alamarBlue assay and image analysis respectively. The reduction of bacterial viability reflected by the restricted metabolic activity of biofilm in our study demonstrated a similar trend as the biomass decrease observed by Jardeleza *et al*, although direct comparison were not possible.

It is also noteworthy to point out that although +ULV displayed the most effective penetration to *P*. *aeruginosa* biofilm, there were no significant differences when comparing the relative viability after five-minute liposomal treatment with the control. The ongoing viability of the biofilm with altered structure reflected its refractory nature. Nevertheless, at 24-hour exposure, +ULV was the most effective formulation to inhibite the *P*. *aeruginosa* biofilm. Indeed, the cationic liposomes lowered the relative viability more than their anionic counterparts, confirming that the liposomal toxicity against bacteria was correlated with the amount of attached nanocarriers within a longer incubation time. The observed inhibition of blank liposomes against both *S*. *aureus* and *P*. *aeruginosa* biofilms can be expected to enhance their anti-biofilm effectiveness if anti-biofilm drugs are encapsulated in these liposomes.

Further studies are needed to evaluate whether multi-species biofilm growing on nasal mucosa rather than on glass or plastic surface behave in a similar way as biofilms in *in vitro* experiments. It has been reported that *in vivo* biofilms possess a few structural and component characteristics that differ from most *in vitro* biofilms[31, 46]. Whether these differences alter biofilms’ physicochemical properties that determine their interactions with liposomes remains unknown. Biofilms found in the nasal sinuses of CRS patients are mixed with the mucus which can trap nanoparticles by adhesion and/or obstruction[47]. To develop nanoparticles that may penetrate through this mucus barrier and avoid the natural mucus clearance mechanism will be the subject of future studies.

## Conclusion

This study showed that the charge and size of liposomes influenced their distribution in biofilms and their intrinsic anti-biofilm effect. Reducing the size of liposomes and formulating liposomes as positively charged enhanced the penetration and inhibition of *S*. *aureus* and *P*. *aeruginosa* biofilms.

## Supporting Information

S1 Fig Influence of liposomes to alamarBlue. This experiment was conducted to confirm the alamarBlue assay was suitable for the liposomal anti-biofilm study. First, 20μl/well alamarBlue was added to wells containing 180μl different liposomes (0.9% saline as the control) in a 96 well clear-bottom microplates and incubated at 37°C for one hour. Then the fluorescence intensity was measured by FLUOstar OPTIMA plate reader (BMG Labtech, Vic, Australia) equipped with an excitation filter of 520–540 nm and an emission filter of 580–600 nm. This test for each liposome was carried out in triplicate and the experiments repeated twice. As result, the fluorescence intensity of alamarBlue treated by +MLV, +ULV,-MLV,-ULV and the control was 874.0 (871.0–880.0), 881.5 (869.0–903.0), 876.0 (869.0–891.0), 876.5 (870.0–882.0), and 886.0 (880.0–893.0) respectively, showing there were no statistical differences between liposomes (Kruskal-Wallis test, *P*>0.05). Therefore it was concluded that effect of liposomes to alamarBlue was negligible. +MLV: cationic multilamellar vesicle; +ULV: cationic unilamellar vesicle;-MLV: anionic multilamellar vesicle;-ULV: anionic unilamellar vesicle.
(TIF)

## References

[1] Boase S, Foreman A, Cleland E, Tan L, Melton-Kreft R, Pant H, et al The microbiome of chronic rhinosinusitis: culture, molecular diagnostics and biofilm detection. BMC infectious diseases. 2013;13:210 10.1186/1471-2334-13-210 23656607 PMC3654890

[2] Dong D, Yulin Z, Xiao W, Hongyan Z, Jia L, Yan X, et al Correlation between bacterial biofilms and osteitis in patients with chronic rhinosinusitis. The Laryngoscope. 2014;124(5):1071–7. Epub 2013/10/12. 10.1002/lary.24424 24114791

[3] Foreman A, Jervis-Bardy J, Wormald PJ. Do biofilms contribute to the initiation and recalcitrance of chronic rhinosinusitis? The Laryngoscope. 2011;121(5):1085–91. 10.1002/lary.21438 21520128

[4] Foreman A, Boase S, Psaltis A, Wormald PJ. Role of bacterial and fungal biofilms in chronic rhinosinusitis. Current allergy and asthma reports. 2012;12(2):127–35. 10.1007/s11882-012-0246-7 22322439

[5] Li H, Wang D, Sun X, Hu L, Yu H, Wang J. Relationship between bacterial biofilm and clinical features of patients with chronic rhinosinusitis. European archives of oto-rhino-laryngology: official journal of the European Federation of Oto-Rhino-Laryngological Societies. 2012;269(1):155–63. 10.1007/s00405-011-1683-y 21739098

[6] Singhal D, Foreman A, Jervis-Bardy J, Wormald PJ. Staphylococcus aureus biofilms: Nemesis of endoscopic sinus surgery. The Laryngoscope. 2011;121(7):1578–83. 10.1002/lary.21805 21647904

[7] Bendouah Z, Barbeau J, Hamad WA, Desrosiers M. Biofilm formation by Staphylococcus aureus and Pseudomonas aeruginosa is associated with an unfavorable evolution after surgery for chronic sinusitis and nasal polyposis. Otolaryngology—head and neck surgery: official journal of American Academy of Otolaryngology-Head and Neck Surgery. 2006;134(6):991–6. 10.1016/j.otohns.2006.03.001 16730544

[8] Jardeleza C, Rao S, Thierry B, Gajjar P, Vreugde S, Prestidge CA, et al Liposome-encapsulated ISMN: a novel nitric oxide-based therapeutic agent against Staphylococcus aureus biofilms. PloS one. 2014;9(3):e92117 10.1371/journal.pone.0092117 24658315 PMC3962386

[9] Ha KR, Psaltis AJ, Butcher AR, Wormald PJ, Tan LW. In vitro activity of mupirocin on clinical isolates of Staphylococcus aureus and its potential implications in chronic rhinosinusitis. The Laryngoscope. 2008;118(3):535–40. 10.1097/MLG.0b013e31815bf2e3 18090864

[10] Fresta M, Puglisi G, Giammona G, Cavallaro G, Micali N, Furneri PM. Pefloxacine mesilate- and ofloxacin-loaded polyethylcyanoacrylate nanoparticles: characterization of the colloidal drug carrier formulation. Journal of pharmaceutical sciences. 1995;84(7):895–902. 7562444 10.1002/jps.2600840721

[11] Montagna MT, Lovero G, Coretti C, De Giglio O, Martinelli D, Bedini A, et al In vitro activities of amphotericin B deoxycholate and liposomal amphotericin B against 604 clinical yeast isolates. Journal of medical microbiology. 2014;63(Pt 12):1638–43. 10.1099/jmm.0.075507-0 25210203 PMC4250836

[12] Fresta M, Furneri PM, Mezzasalma E, Nicolosi VM, Puglisi G. Correlation of trimethoprim and brodimoprim physicochemical and lipid membrane interaction properties with their accumulation in human neutrophils. Antimicrobial agents and chemotherapy. 1996;40(12):2865–73. 9124856 10.1128/aac.40.12.2865 PMC163637

[13] Hamblin KA, Wong JP, Blanchard JD, Atkins HS. The potential of liposome-encapsulated ciprofloxacin as a tularemia therapy. Frontiers in cellular and infection microbiology. 2014;4:79 10.3389/fcimb.2014.00079 24995163 PMC4062069

[14] Furneri PM, Fresta M, Puglisi G, Tempera G. Ofloxacin-loaded liposomes: in vitro activity and drug accumulation in bacteria. Antimicrobial agents and chemotherapy. 2000;44(9):2458–64. 10952595 10.1128/aac.44.9.2458-2464.2000 PMC90085

[15] Meers P, Neville M, Malinin V, Scotto AW, Sardaryan G, Kurumunda R, et al Biofilm penetration, triggered release and in vivo activity of inhaled liposomal amikacin in chronic Pseudomonas aeruginosa lung infections. The Journal of antimicrobial chemotherapy. 2008;61(4):859–68. 10.1093/jac/dkn059 18305202

[16] Obonyo M, Zhang L, Thamphiwatana S, Pornpattananangkul D, Fu V, Zhang L. Antibacterial activities of liposomal linolenic acids against antibiotic-resistant Helicobacter pylori. Molecular pharmaceutics. 2012;9(9):2677–85. 10.1021/mp300243w 22827534 PMC3433584

[17] Lai SK, Suk JS, Pace A, Wang YY, Yang M, Mert O, et al Drug carrier nanoparticles that penetrate human chronic rhinosinusitis mucus. Biomaterials. 2011;32(26):6285–90. 10.1016/j.biomaterials.2011.05.008 21665271 PMC3130096

[18] Forier K, Raemdonck K, De Smedt SC, Demeester J, Coenye T, Braeckmans K. Lipid and polymer nanoparticles for drug delivery to bacterial biofilms. Journal of controlled release: official journal of the Controlled Release Society. 2014;190:607–23. 10.1016/j.jconrel.2014.03.055 24794896

[19] Ahmed K, Gribbon PN, Jones MN. The application of confocal microscopy to the study of liposome adsorption onto bacterial biofilms. Journal of liposome research. 2002;12(4):285–300. 10.1081/LPR-120016195 12519626

[20] Forier K, Messiaen AS, Raemdonck K, Nelis H, De Smedt S, Demeester J, et al Probing the size limit for nanomedicine penetration into Burkholderia multivorans and Pseudomonas aeruginosa biofilms. Journal of controlled release: official journal of the Controlled Release Society. 2014;195:21–8. 10.1016/j.jconrel.2014.07.061 25125326

[21] Allison DG. The biofilm matrix. Biofouling. 2003;19(2):139–50. 10.1080/0892701031000072190 14618698

[22] Bangham AD, Standish MM, Weissmann G. The action of steroids and streptolysin S on the permeability of phospholipid structures to cations. Journal of molecular biology. 1965;13(1):253–9. 5859040 10.1016/s0022-2836(65)80094-8

[23] Hinna A, Steiniger F, Hupfeld S, Stein P, Kuntsche J, Brandl M. Filter-extruded liposomes revisited: a study into size distributions and morphologies in relation to lipid-composition and process parameters. Journal of liposome research. 2015:1–10. 10.3109/08982104.2015.1022556 25826203

[24] Parmentier J, Thomas N, Mullertz A, Fricker G, Rades T. Exploring the fate of liposomes in the intestine by dynamic in vitro lipolysis. International journal of pharmaceutics. 2012;437(1–2):253–63. 10.1016/j.ijpharm.2012.08.018 22939968

[25] Honig MG, Hume RI. Dil and diO: versatile fluorescent dyes for neuronal labelling and pathway tracing. Trends in neurosciences. 1989;12(9):333–5, 40–1. 2480673

[26] Andar AU, Hood RR, Vreeland WN, Devoe DL, Swaan PW. Microfluidic preparation of liposomes to determine particle size influence on cellular uptake mechanisms. Pharmaceutical research. 2014;31(2):401–13. 10.1007/s11095-013-1171-8 24092051

[27] Merritt JH, Kadouri DE, O'Toole GA. Growing and analyzing static biofilms. Current protocols in microbiology. 2005;Chapter 1:Unit 1B 10.1002/9780471729259.mc01b01s00 PMC4568995 18770545

[28] Peeters E, Nelis HJ, Coenye T. Comparison of multiple methods for quantification of microbial biofilms grown in microtiter plates. Journal of microbiological methods. 2008;72(2):157–65. 10.1016/j.mimet.2007.11.010 18155789

[29] Cottenye N, Cui ZK, Wilkinson KJ, Barbeau J, Lafleur M. Interactions between non-phospholipid liposomes containing cetylpyridinium chloride and biofilms of Streptococcus mutans: modulation of the adhesion and of the biodistribution. Biofouling. 2013;29(7):817–27. 10.1080/08927014.2013.807505 23826726

[30] Sandt C, Barbeau J, Gagnon MA, Lafleur M. Role of the ammonium group in the diffusion of quaternary ammonium compounds in Streptococcus mutans biofilms. The Journal of antimicrobial chemotherapy. 2007;60(6):1281–7. 10.1093/jac/dkm382 17932074

[31] Flemming HC, Neu TR, Wozniak DJ. The EPS matrix: the "house of biofilm cells". Journal of bacteriology. 2007;189(22):7945–7. 10.1128/JB.00858-07 17675377 PMC2168682

[32] Xu X, Khan MA, Burgess DJ. A quality by design (QbD) case study on liposomes containing hydrophilic API: I. Formulation, processing design and risk assessment. International journal of pharmaceutics. 2011;419(1–2):52–9. 10.1016/j.ijpharm.2011.07.012 21787854

[33] Gross M, Cramton SE, Gotz F, Peschel A. Key role of teichoic acid net charge in Staphylococcus aureus colonization of artificial surfaces. Infection and immunity. 2001;69(5):3423–6. 10.1128/IAI.69.5.3423-3426.2001 11292767 PMC98303

[34] Peschel A, Otto M, Jack RW, Kalbacher H, Jung G, Gotz F. Inactivation of the dlt operon in Staphylococcus aureus confers sensitivity to defensins, protegrins, and other antimicrobial peptides. The Journal of biological chemistry. 1999;274(13):8405–10. 10085071 10.1074/jbc.274.13.8405

[35] Grant SS, Hung DT. Persistent bacterial infections, antibiotic tolerance, and the oxidative stress response. Virulence. 2013;4(4):273–83. 10.4161/viru.23987 23563389 PMC3710330

[36] Rehman ZU, Wang Y, Moradali MF, Hay ID, Rehm BH. Insights into the assembly of the alginate biosynthesis machinery in Pseudomonas aeruginosa. Applied and environmental microbiology. 2013;79(10):3264–72. 10.1128/AEM.00460-13 23503314 PMC3685254

[37] Sonohara R, Muramatsu N, Ohshima H, Kondo T. Difference in surface properties between Escherichia coli and Staphylococcus aureus as revealed by electrophoretic mobility measurements. Biophysical chemistry. 1995;55(3):273–7. 7626745 10.1016/0301-4622(95)00004-h

[38] Powell LC, Pritchard MF, Emanuel C, Onsoyen E, Rye PD, Wright CJ, et al A nanoscale characterization of the interaction of a novel alginate oligomer with the cell surface and motility of Pseudomonas aeruginosa. American journal of respiratory cell and molecular biology. 2014;50(3):483–92. 10.1165/rcmb.2013-0287OC 24074505

[39] Sudagidan M, Erdem I, Cavusoglu C, Ciftcloglu M. Investigation of the surface properties of Staphylococcus epidermidis strains isolated from biomaterials. Mikrobiyoloji bulteni. 2010;44(1):93–103. 20455404

[40] van Merode AE, Pothoven DC, van der Mei HC, Busscher HJ, Krom BP. Surface charge influences enterococcal prevalence in mixed-species biofilms. Journal of applied microbiology. 2007;102(5):1254–60. 10.1111/j.1365-2672.2006.03187.x 17448160

[41] Rogers SA, Huigens RW III, Cavanagh J, Melander C. Synergistic effects between conventional antibiotics and 2-aminoimidazole-derived antibiofilm agents. Antimicrobial agents and chemotherapy. 2010;54(5):2112–8. 10.1128/AAC.01418-09 20211901 PMC2863642

[42] Stefaan J. Soenena PR-G, Montenegro JM, Parakb WJ, De Smedta SC, Braeckmansa K. Cellular toxicity of inorganic nanoparticles: Common aspects and guidelines for improved nanotoxicity evaluation. nanotoday. 2011;6(5):19 10.1016/j.nantod.2011.08.001

[43] Hajipour MJ, Fromm KM, Ashkarran AA, Jimenez de Aberasturi D, de Larramendi IR, Rojo T, et al Antibacterial properties of nanoparticles. Trends in biotechnology. 2012;30(10):499–511. 10.1016/j.tibtech.2012.06.004 22884769

[44] Nel AE, Madler L, Velegol D, Xia T, Hoek EM, Somasundaran P, et al Understanding biophysicochemical interactions at the nano-bio interface. Nature materials. 2009;8(7):543–57. 10.1038/nmat2442 19525947

[45] Desbois AP, Smith VJ. Antibacterial free fatty acids: activities, mechanisms of action and biotechnological potential. Applied microbiology and biotechnology. 2010;85(6):1629–42. 10.1007/s00253-009-2355-3 19956944

[46] Bjarnsholt T, Alhede M, Alhede M, Eickhardt-Sorensen SR, Moser C, Kuhl M, et al The in vivo biofilm. Trends in microbiology. 2013;21(9):466–74. 10.1016/j.tim.2013.06.002 23827084

[47] Lai SK, Wang YY, Hanes J. Mucus-penetrating nanoparticles for drug and gene delivery to mucosal tissues. Advanced drug delivery reviews. 2009;61(2):158–71. 10.1016/j.addr.2008.11.002 19133304 PMC2667119

