# Supplementary figures and images for: Distribution and Inhibition of Liposomes on Staphylococcus aureus and Pseudomonas aeruginosa Biofilm
Source: PLoS One. 2015 Jun 30;10(6):e0131806. doi: 10.1371/journal.pone.0131806 (PMC4488362; doi:10.1371/journal.pone.0131806)

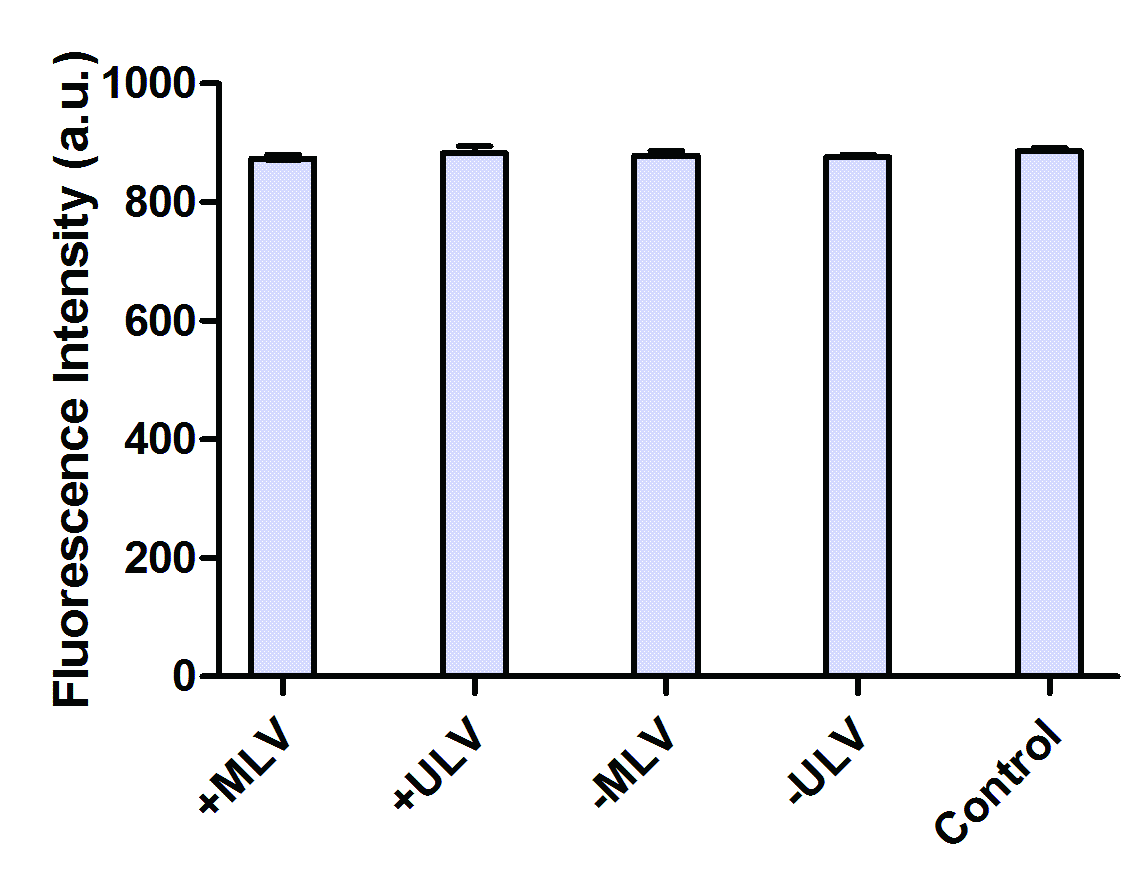

Supplement: S1 Fig — This experiment was conducted to confirm the alamarBlue assay was suitable for the liposomal anti-biofilm study. First, 20μl/well alamarBlue was added to wells containing 180μl different liposomes (0.9% saline as the control) in a 96 well clear-bottom microplates and incubated at 37°C for one hour. Then the fluorescence intensity was measured by FLUOstar OPTIMA plate reader (BMG Labtech, Vic, Australia) equipped with an excitation filter of 520–540 nm and an emission filter of 580–600 nm. This test for each liposome was carried out in triplicate and the experiments repeated twice. As result, the fluorescence intensity of alamarBlue treated by +MLV, +ULV,-MLV,-ULV and the control was 874.0 (871.0–880.0), 881.5 (869.0–903.0), 876.0 (869.0–891.0), 876.5 (870.0–882.0), and 886.0 (880.0–893.0) respectively, showing there were no statistical differences between liposomes (Kruskal-Wallis test, P>0.05). Therefore it was concluded that effect of liposomes to alamarBlue was negligible. +MLV: cationic multilamellar vesicle; +ULV: cationic unilamellar vesicle;-MLV: anionic multilamellar vesicle;-ULV: anionic unilamellar vesicle. (TIF) [file pone.0131806.s001.tif]
